# Internal Carotid Artery Dissection With Thrombosis in a Child With Prothrombin Gene Mutation

**DOI:** 10.7759/cureus.35481

**Published:** 2023-02-26

**Authors:** Victor N Oboli, Arisa Poudel, Muhammad Waseem

**Affiliations:** 1 Pediatrics, NYC (New York City) Health + Hospitals/Lincoln, New York City, USA; 2 Emergency Medicine, NYC (New York City) Health + Hospitals/Lincoln, New York City, USA

**Keywords:** arterial stroke, venous thrombosis, arterial thrombosis, prothrombin gene mutation, stroke

## Abstract

Prothrombin gene mutation (prothrombin thrombophilia) is an inherited disorder that increases the risk of venous thrombosis. However, limited data exist on the risk of arterial stroke in an at-risk population. Several meta-analyses report slightly increased risk in specific populations. We report a 10-year-old Hispanic girl who presented to the emergency department with a seizure. This seizure occurred five days after she tripped and fell without any initial associated symptoms. She had left-sided hemiparesis on physical examination after the seizure. Imaging revealed internal carotid artery (ICA) dissection with thrombus, right caudate nucleus and putamen infarcts, and ischemic penumbra. She subsequently had an endovascular thrombectomy of the right ICA with reperfusion. Genetic testing showed a prothrombin gene mutation (G20210A). Prothrombin gene mutation was the most likely explanation for her stroke in the absence of a significant risk factor for arterial thrombosis or an underlying hypercoagulable disorder. Further investigations are required to determine the risks and evaluate the correlation between prothrombin gene mutation and ischemic stroke in children.

## Introduction

Prothrombin, also known as factor II, is thrombin's precursor, leading to fibrin and thrombus formation in the coagulation cascade. After factor V mutation, prothrombin gene mutation is the second most common congenital inherited thrombophilia [1,2]. Prothrombin gene mutation arises when the gene responsible for making prothrombin is defective. For example, single-point mutations in the gene coding for prothrombin (factor II: A20210) will increase the risk of thromboembolism, predominantly venous rather than arterial [1,3,4]. A stroke is an abrupt onset of neurological deterioration by manifestations of deficits, and several risk factors are associated with the development of arterial stroke, including prothrombin gene mutation [5].

The point mutation at G20210A in the prothrombin gene results from substituting adenine (A) for guanine (G) at position 20210 in a non-coding region [6,7]. Most individuals with this pathogenic variant inheritance are heterozygous in origin, although homozygosity has been reported in a small percentage of patients [2,7,8]. Heterozygous defective prothrombin gene mutations occur in about 2-4% of Caucasians and are more common in individuals of European ancestry. The mutation is uncommon in African Americans (approximately 0.5%) and rare in Asians, Africans, Hispanics, and Native Americans. The homozygous form is regarded as uncommon, with a typical occurrence of roughly one in 10 000 individuals [7]. The gene mutation is equally as common in men and women. There is no sexual predilection and no association has been demonstrated between this mutation and an individual's blood group [2,3]. However, most individuals with a prothrombin gene mutation will never develop blood clots or thromboembolic events in their entire lifetime. Any individuals with the prothrombin gene mutation that develops a blood clot usually have additional risk factors. Therefore, recognizing additional risk factors for developing arterial stroke in a patient is crucial.

Common risk factors include trauma, prior thromboembolism, surgery, hospitalization, oral contraceptive pills (OCPs), long-distance air travel, pregnancy, or immobility [1,9].

## Case presentation

**Figure 1:**
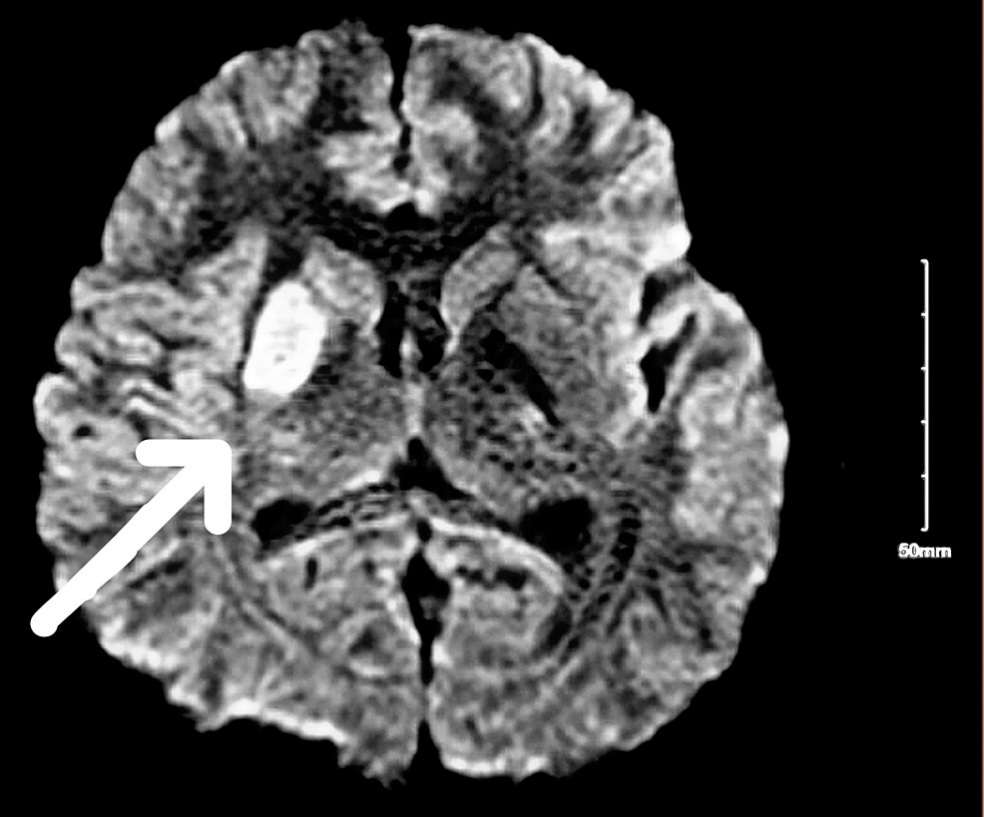
Magnetic resonance imaging of the brain showing acute right caudate nucleus and putamen infarcts with suggestion of faint petechial hemorrhage with mild mass effect and partial effacement of the frontal horn of the right lateral ventricle (white arrow). mm - millimeter

**Figure 2:**
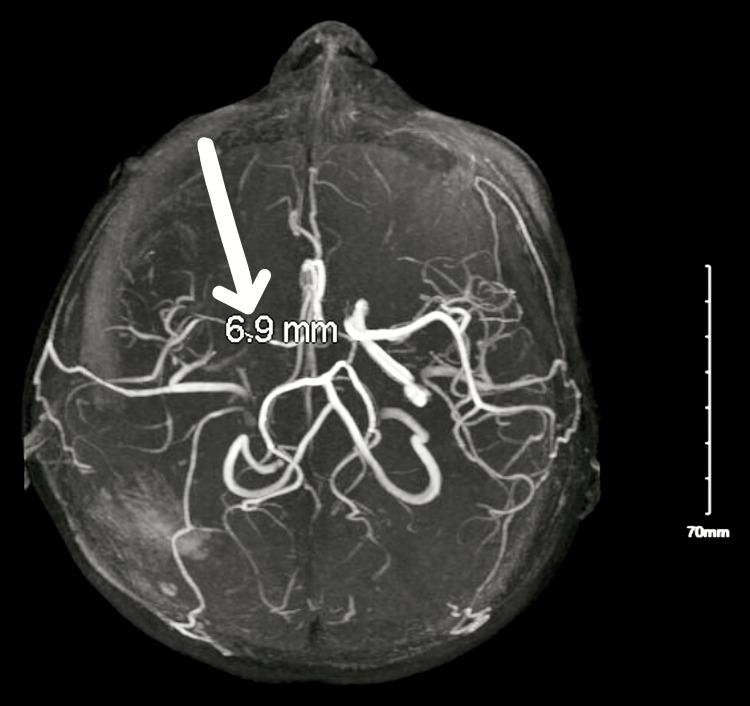
Magnetic resonance angiography of the brain showing markedly attenuated flow-related enhancement of the right middle cerebral artery, with likely opacification through the anterior communicating and/or posterior communicating arteries, with segmental lack of flow (6.9 mm) related enhancement in the M1 segment concerning for presence of thrombus (white arrow). mm - millimeter

A previously healthy 10-year-old female presented to the emergency department (ED) because of a new onset seizure. Her parent reported a minor head injury five days prior to this presentation. On arrival, there was a loss of the right gaze, right facial twitching, and right-sided extensor posturing. After stabilization, a non-contrast head computerized tomography (CT) scan was negative for abnormalities. She got transferred to a tertiary hospital for a video electroencephalogram (EEG). A few hours later, she developed left-sided hemiparesis. Emergent brain magnetic resonance imaging (MRI), magnetic resonance angiography (MRA), and CT angiography (CTA) of the head and neck depicted findings consistent with right ICA dissection with thrombus, caudate/putamen infarcts, and small petechial hemorrhage (Figures 1, 2). She subsequently had a successful endovascular thrombectomy of the right distal ICA with thrombolysis in cerebral infarction (TICI) 3 reperfusion. She received anticoagulation therapy and antiepileptics after surgery. A large right hemispheric acute infarct noted on a non-contrast brain MRI complicated her recovery course following the worsening of her left upper extremity weakness.

Further investigations revealed positive antinuclear antibodies (1:80 with speckled antinuclear antibodies (ANA) detected by indirect immunofluorescence assay (IFA)), mildly increased antithrombin activity (137%, reference range: 90-131%), and factor 8 activity (165%, reference range: 53-131%). In addition, genetic testing revealed pathogenic variant C of prothrombin gene mutation (97g>A; G20210A). She gradually made a significant recovery with her motor and gait functions following medical and surgical interventions, but intermittent headaches, left-sided body weakness, and dysarthria persisted. Nevertheless, she resumed her regular activities of daily living, including school attendance and follow-up examinations.

## Discussion

Stroke in children is a rare disease. It is an infrequent neurological emergency in children but a significant cause of neurological morbidity. It is often under-recognized, although up to 10-15% of children present to the ED with neurological manifestations [10]. Stroke being uncommon, pediatric patients presenting with such symptoms are more likely to experience delays in recognizing and diagnosing. However, prompt recognition is essential for an optimal outcome.

Stroke in children is associated with diverse risk factors (Table 1). In addition, the causes and risk factors of childhood stroke differ from adults. It can result from primary vascular disease, bleeding disorder, or secondary disorders that can cause thrombotic or embolic occlusions.

**Table 1:** Risk factors for stroke in children. Adapted from Freundlich et al., 2012 [11].

Cardiac	Hematologic disorders	Vasculitis	Infection	Metabolic	Drugs
Congenital heart diseases	Hemoglobinopathies such as sickle cell disease	Systemic lupus erythematosus (SLE)	Human immunodeficiency virus infection (HIV)	Homocystinuria	Cocaine
Arrhythmias	Hypercoagulable disorders (Protein C and S deficiency, antithrombin deficiency, prothrombin G20210A variant)	Kawasaki disease	Syphilis – neurosyphilis	Hyperlipidemia	Amphetamines
Rheumatic heart disease	Hyperviscosity states (leukemia, hyperproteinemia, thrombocytosis, polycythemia)			Mitochondrial disorders	
	Coagulation cascade disorders				

The incidence of stroke has increased several folds in children with sickle cell disease (SCD), and children with cyanotic heart disease are also at high risk for stroke [12]. In addition, some children develop strokes from vasculitis due to purulent meningitis as a superinfection [13]. Several mutations may be associated with stroke, of which prothrombin gene mutation is one [14]. Arterial ischemic stroke in children with coronavirus disease 2019 (COVID-19) has been reported [14-16]. In recent years, COVID-19 has improved our understanding of stroke in children [17]. It is believed that COVID-19 directly affects vascular endothelium through interactions with angiotensin-converting enzyme (ACE) receptors [18].

Although prothrombin gene mutation (G20210A) is an important cause of venous thrombosis, its role in arterial thrombosis remains uncertain. It is inherited in an autosomal dominant pattern [19], and causes an increased production of prothrombin. It is crucial to identify the presence of genetic mutations in a pediatric patient presenting with stroke to provide prompt treatment, prevent recurrences, and educate on the risk of thrombosis in family members [20]. The signs and symptoms may be vague and non-specific in children; hence, it is difficult to pinpoint the etiology. Presenting symptoms and signs majorly depends on the stroke type and the brain region involved. The localizing signs and symptoms such as hemiparesis or hemifacial weakness, speech or language dysfunctions, vision disturbances, or ataxia occur in children with stroke. A stroke should be considered as a possible diagnosis within the differential for patients who present with new neurological findings. Children who are suspected of having a stroke should undergo urgent neuroimaging. MRI is considered an ideal method to evaluate suspected stroke in pediatric age. The initial evaluation should emphasize supportive measures, such as airway stabilization, administration of oxygen, maintenance of euglycemia, and treatment of seizures if they are present.

## Conclusions

Our case report depicts the significance of considering trauma as a predisposing factor for developing arterial stroke in a patient with prothrombin gene deletion. Nonetheless, more longitudinal studies will be needed to correlate the relationship between developing arterial thrombus in patients with prothrombin gene deletion and several associated risk factors. In addition, diagnosis of stroke in children is challenging; we recommend that pediatric emergency physicians consider and have a high index of suspicion and be familiar with stroke risk factors. Finally, there is also a need for urgent neuroimaging to confirm its diagnosis and the role of reperfusion therapies.
